# Transit Amplifying Cells (TACs): a still not fully understood cell population

**DOI:** 10.3389/fbioe.2023.1189225

**Published:** 2023-05-09

**Authors:** Ranieri Cancedda, Maddalena Mastrogiacomo

**Affiliations:** ^1^ Emeritus Professor, Università degli Studi di Genova, Genoa, Italy; ^2^ Dipartimento di Medicina Interna e Specialità Mediche (DIMI), Università Degli Studi di Genova, Genova, Italy

**Keywords:** tissue differentiation, tissue regeneration, cell dedifferentiation, cell reprogramming, stem cells, cultured cells, hair follicle, intestinal crypt

## Abstract

Maintenance of tissue homeostasis and tissue regeneration after an insult are essential functions of adult stem cells (SCs). In adult tissues, SCs proliferate at a very slow rate within “stem cell niches”, but, during tissue development and regeneration, before giving rise to differentiated cells, they give rise to multipotent and highly proliferative cells, known as transit-amplifying cells (TACs). Although differences exist in diverse tissues, TACs are not only a transitory phase from SCs to post-mitotic cells, but they also actively control proliferation and number of their ancestor SCs and proliferation and differentiation of their progeny toward tissue specific functional cells. Autocrine signals and negative and positive feedback and feedforward paracrine signals play a major role in these controls. In the present review we will consider the generation and the role played by TACs during development and regeneration of lining epithelia characterized by a high turnover including epidermis and hair follicles, ocular epithelial surfaces, and intestinal mucosa. A comparison between these different tissues will be made. There are some genes and molecular pathways whose expression and activation are common to most TACs regardless their tissue of origin. These include, among others, Wnt, Notch, Hedgehog and BMP pathways. However, the response to these molecular signals can vary in TACs of different tissues. Secondly, we will consider cultured cells derived from tissues of mesodermal origin and widely adopted for cell therapy treatments. These include mesenchymal stem cells and dedifferentiated chondrocytes. The possible correlation between cell dedifferentiation and reversion to a transit amplifying cell stage will be discussed.

## Introduction

Physiological turnover and insult damage repair of body tissues are made possible by the presence of stem cells (SCs). In adult tissues, SCs proliferate at very slow rate within “stem cell niches”, but, during tissue development and regeneration, before giving rise to differentiated cells, they change first to a multipotent and more proliferative state, known as transit-amplifying cells (TACs). Indeed, TACs can be defined as a non-differentiated, proliferating cell population in transition between SCs and differentiated cells. In adult tissues with a high turn-over, SCs fluctuate from a quiescent state to a cell division once every 1–5 days while TACs are characterized by shorter doubling times. A hematopoietic SC was first described by Till and Mc Culloch in 1961 (Till and McCulloch, 2011) and the stem cell niche was first suggested in 1978 by Schofield to describe local environments required for the maintenance of hematopoietic cell stemness (Schofield, 1978). Likewise, TACs and negative feedback in a vertebrate stem cell system were first described in hematopoiesis, where, when a deficiency of red blood cells, white blood cells or platelets is sensed, progenitor cells are activated and proliferate to restore the correct number of cells of the different lineages. Several excellent reviews already exist on hematopoietic SCs and hematopoiesis control. Examples of these reviews are (Shizuru et al., 2005; Orkin and Zon, 2008; Seita and Weissman, 2010; Pinho and Frenette, 2019). In the present review we will consider SCs and SC progeny during development and regeneration of solid tissues.

## Holoclones, meroclones, and paraclones: SC and TAC identification in epithelial tissues

Solid tissue TACs were first described in cultures of cells from epidermis. In their 1987 seminal paper, Yann Barrandon and Howard Green reported the existence of three different types of clones in primary cultures of skin epidermal cells: holoclones, meroclones, and paraclones (Barrandon and Green, 1987). The relative percentage of the different clone types varies with the epidermis donor age (De Luca et al., 2006). Young donors give rise to a higher percentage of holoclones and a lower number of paraclones than old donors. Holoclones have the greatest reproductive capacity, whereas paraclones are cells with a short replicative lifespan. Meroclones were defined by the authors as a transitional stage between holoclones and paraclones and are characterized by an intermediate proliferation rate between holoclones and paraclones. A key observation was that during subculture transfers, holoclones became meroclones, and progressively converted to paraclones, suggesting a directional growth potential restriction. Over the years, Barrandon’s and De Luca-Pellegrini’s groups have demonstrated that holoclone-forming cells have the characteristics of SCs and meroclones and paraclones have properties expected for TACs. Meroclones are considered “young” TACs endowed with a greater proliferative capacity than paraclones (Rochat et al., 1994; Pellegrini et al., 1999a; Pellegrini et al., 2001). The extensive proliferative potential of holoclones, the possibility to generate a mature epithelium from a single holoclone eventually transplanted, obtaining a mature epithelium with the distinct cellular lineages after the transplant, in addition to the permanent epithelium regeneration in patients transplanted with autologous cultured epithelial grafts, provided compelling evidence of the stem nature of holoclones and that keratinocyte “stemness” can be preserved *in vitro* when using proper culture conditions (Gallico et al., 1984; Pellegrini et al., 1997; Pellegrini et al., 1999b). The clinical success of Advanced Therapy Medicinal Products (ATMPs) based on *in vitro* obtained epithelia requires the presence of SCs in the grafts because of the continuous tissue renewal *in vivo*. The most common trustworthy method to confirm the presence and the number of SCs in an epidermal culture is still the identification of holoclones in the cell culture.

The epidermis is a stratified squamous epithelium forming the barrier protecting from outside microbes and retaining body fluids. Lechler and Fuchs showed that *in vivo* the epidermis stratification occurs through asymmetric cell divisions in which basal epidermal cells use their polarity to divide asymmetrically, generating a committed suprabasal cell and a proliferative basal cell (SC or TAC) (Lechler and Fuchs, 2005). They further demonstrated differences in the integrins and cadherins distribution and that these proteins are essential to align the mitotic spindle parallel to the basal membrane. However, this model was challenged by other authors which proposed that SCs divide symmetrically and stochastically adopt SC or TAC fates (Jones et al., 2007) Based on differences in integrin function and expression, an initial separation of human epidermal SCs from TACs was attempted by Jones and Watts (Jones and Watt, 1993). Keratinocytes with characteristics of stem cells were isolated to greater than 90% purity from cultured human epidermis based on the high surface expression of beta-1 integrin and the rapid adhesion to extracellular matrix (ECM) proteins. Proliferating keratinocytes that adhered to the ECM more slowly had characteristics of TACs and after 1 to 5 divisions, all their daughter cells underwent terminal differentiation. Presently, the clonal detection of a high level of nuclear expression of the p63 transcription factor, a p53 homologue essential for regenerative proliferation in epithelial development, is probably the best method to distinguish human keratinocyte stem cells from their TAC progeny. P63 alpha isoform is abundantly expressed by holoclones, but is undetectable in paraclones (Pellegrini et al., 2001). TA keratinocytes, immediately after their withdrawal from the stem cell compartment (i.e., meroclones), have greatly reduced p63 alpha, even though they possess very appreciable proliferative capacity. However, it was reported that *in vivo* the immediately supra-basal cells (early TACs) present the higher amount of both phosphorylated and non-phosphorylated p63 (Suzuki and Senoo, 2012). Indeed, the presence of p63 is crucial for the correct epidermis development. Mice lacking p63 are born alive, but their skin does not progress past an early developmental stage: it lacks stratification, does not express differentiation markers and structures such as hair follicles, teeth, and mammary glands, are absent (Mills et al., 1999). Generation of TACs from SCs is promoted by the sigma isoform of the 14-3-3 protein family. Downregulation of 14-3-3 sigma resulted in keratinocytes remaining in the stem cell compartment and maintaining p63 nuclear expression during serial cultivation (Pellegrini et al., 2001). The identification of the p63 stem cell marker has been relevant for the clinical application of cultured epithelia as well as for tumorigenesis studies. The Ras family of small GTPases has a key role in skin tumorigenesis. Indeed, activating mutations in Ras genes have been found in human cutaneous squamous cell carcinomas. Dellambra has suggested, although not completely proven, that Ras family members can play a role also in normal transition from epithelial SCs to TACs (Dellambra, 2016). Interestingly, the identification and an initial characterization of “early” and “late” TACs in the human epidermis was recently reported (Lotti et al., 2022). The highly proliferative “early” TAC population, characterized by a rapid adherence to type IV collagen, was isolated from the interfollicular epidermis. Proliferation and colony-forming efficiency of these cells were higher than in “late” TACs characterized by a slow adherence to type IV collagen. Differentiation marker analysis confirmed the unique phenotype of “early TACs (high expression of FOXM1, delta-Np63, Survivin, Ki67).

## Hair follicles: skin appendages with their own individual set of epithelial SCs

During embryogenesis the hair follicle development begins with a local epidermis thickening, named placode. Subsequently, under the epithelial placode, a condensation of mesenchymal cells occurs giving rise to what will be the dermal papilla surrounding the hair follicle (Fuchs, 2007; Driskell et al., 2011). The dermal papilla regulates development of the epidermal follicle development and is dependent upon signals from the epidermis for its development and maintenance. In the follicle development, the Wnt family is the earliest and the most critical regulator for early development of the epidermis (Chen et al., 2012). An intraepidermal Wnt signal is necessary and sufficient for hair follicle initiation. However, the subsequent development depends on reciprocal signaling crosstalk of epidermal and dermal cells (Fu and Hsu, 2013). In mice, Wnt/beta-Catenin signals, such as Wnt10b, are required for initiation, development, and regeneration of the hair follicles (Andl et al., 2002; Wu et al., 2020). Wnt10b was initially expressed uniformly in the epidermis and is markedly upregulated in follicular placodes (Reddy et al., 2001). The formation of placodes that generate the follicles is blocked if the beta-Catenin is mutated during embryogenesis (Huelsken et al., 2001). In the Wnt/beta-Catenin signaling pathway, beta-Catenin is translocated from the cytoplasm to the nucleus, where it interacts with the TCF/LEF transcription factors to activate gene expression. For a review see (Katoh and Katoh, 2007).

**FIGURE 1:**
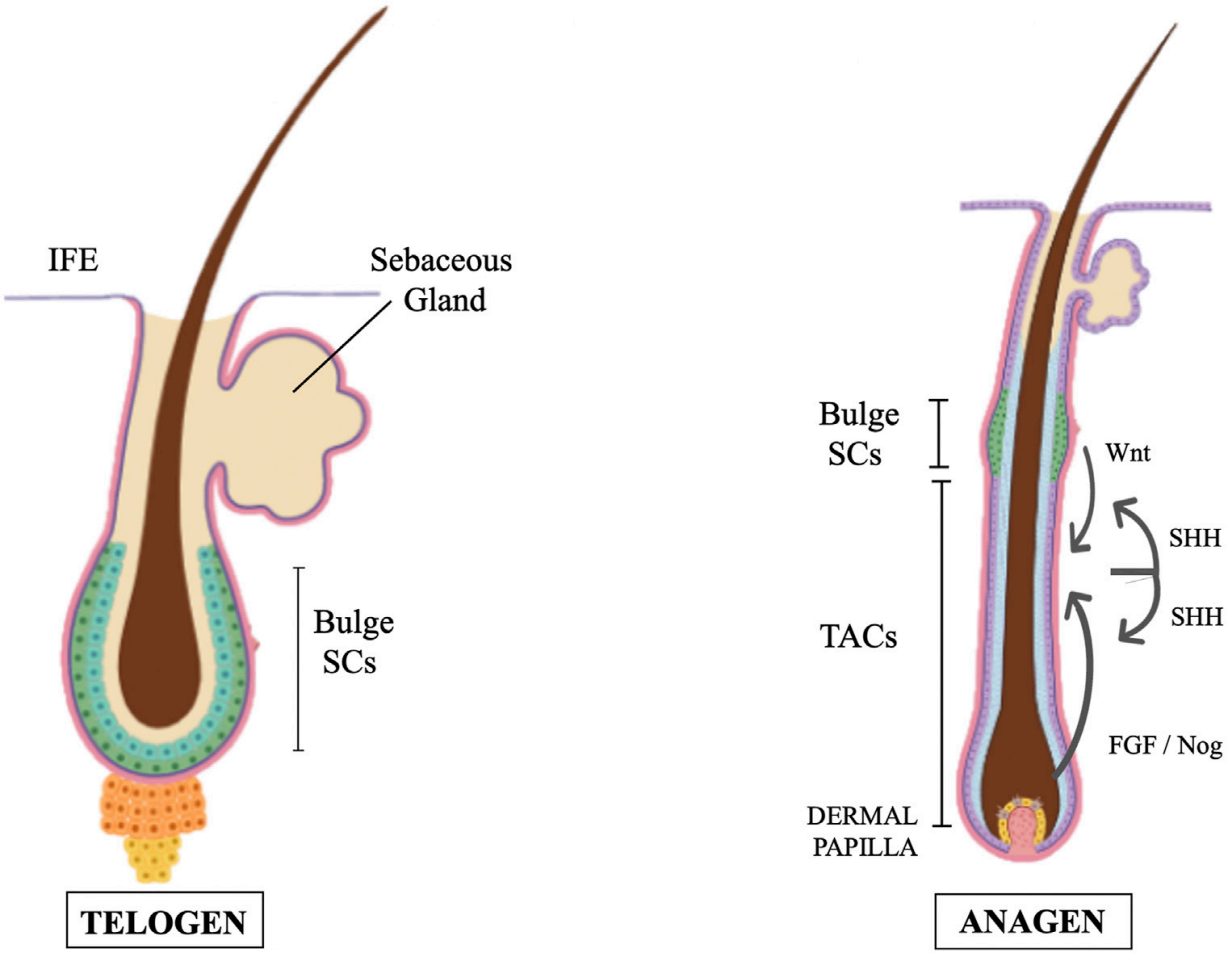
Cartoons of two distinct hair cycle stages (telogen and anagen) showing SCs (greenish cells) and their relative positions. TACs are absent in telogen and are formed at mid-anagen. Feedback circuitry between TACs and SCs play a significant role. TACs contribute to generating the SCs niche and stimulate quiescent SCs to self-renew. Proteins of the Wnt pathway activate the transformation of hair follicle SCs to TACs. While primed SCs generate TACs, quiescent-SCs only proliferate after TACs form and begin expressing Sonic Hedgehog (SHH). SHH also controls several signaling pathways in dermal papilla cells thus regulating the papilla inductive effects on the follicle epithelium by feeding back TACs. IFE = Inter Follicular Epidermis (modification of figure templates created with BioRender.com).

Hair follicles undergo natural regeneration in adult mammals and like in glabrous epidermis, the colony-forming cells from human scalp hair follicles are holoclones (SCs) or meroclones and paraclones (TACs). Each hair follicle is an individual entity, with its own SCs that generate TACs and differentiated cell progeny at well-defined anatomical positions. Moreover, hair follicles cycle between destruction (catagen), rest (telogen), and regrowth (anagen). As such, the hair follicle became a good model to investigate the role of SCs and TACs during mammal tissue regeneration. Several hundred SCs are concentrated in a region which corresponds to the position of the bulge when this anatomical structure can be identified (Figure 1). Some of these SCs have extensive growth potential, as they can undergo at least 130 doublings (Rochat et al., 1994). More specifically, telogen hair follicles contain quiescent SCs located in the bulge while in anagen more activation prone SCs exist which are anatomically located in the hair germ immediately beneath the bulge. TACs are not present in telogen follicles and are an anagen-specific population. Upon anagen entry, TACs are produced by the SCs located in the hair germ. During anagen, TACs proliferate and generate distinct types of differentiated cells. During catagen, TACs are destroyed, and hair follicles are remodeled back to their telogen structure. The presence of true SCs only in the bulge is controversial. Clinical evidence indicates that bulge SCs can regenerate the whole epidermis. In deep burned patients which have lost the skin coverage, the epidermis can reform provided that the hair bulges were not affected. Regardless of this, some authors, based on a careful characterization of cells in the adult mouse hair follicle, suggested the existence of different SC populations located in different regions of the hair follicle and defined by distinct protein expression and gene promoter activity (Schepeler et al., 2014). In our opinion, while we are still searching for specific markers to distinguish between SCs and TACs, the identification and definition of different SC populations and TACs could be seen more as a semantic matter than a real distinction between true SCs and their progeny.

Hair germ SCs and anagen TACs are regulated by a variety of molecular signals released by different cell types. Taking advantage of the mouse hair follicle model the different cell interactions occurring during epidermis and dermis development and wound repair have been deeply investigated. Comprehensive reviews on this aspect are in (Solanas and Benitah, 2013; Zhang and Hsu, 2017). However, it should be here noted that feedback circuitry between TACs and SCs play a significant role in these processes and, more generally, in tissue homeostasis and regeneration. Some studies have shown that epidermal stem cells actively interact with their progeny (Hsu and Fuchs, 2012; Hsu et al., 2014). TACs contribute to the SC niche generation and stimulate quiescent SCs to self-renew and to maintain long-term regenerative capacity. At the same time, they instruct SCs to replenish downstream lineages. Beta catenin, a protein of the Wnt pathway, activates the expression of the nuclear transcription factor Myc and is involved in the transformation of hair follicle SCs to TACs (Shen et al., 2017). Using hair follicles as paradigm, Hsu, Li and Fuchs also showed that while primed SCs generate TACs, quiescent-SCs only proliferate after TACs form and begin expressing Sonic Hedgehog (SHH) (Hsu et al., 2014). TAC generation is independent of autocrine SHH, but their pool wanes if they can’t produce it. This is the consequence of two actions of SHH: to stimulate quiescent-SC proliferation and, by feed-back mechanisms, to trigger dermal mesenchyme to fuel TAC production. The quiescent-SC special sensitivity to SHH signaling is associated to their high expression of the Growth arrest specific 1 (GAS1), a co-receptor for SHH. Without input from quiescent-SCs, replenishment of primed-SCs is compromised, eventually leading to regeneration failure. These findings identify TACs as transient but indispensable players in determining the SC niche and disclose an interdependency of primed and quiescent SCs during tissue regeneration. A subsequent study revealed that TACs, not only orchestrate the generation of their own progeny, but also the of neighboring lineages to achieve a coordinated regeneration of the hair follicles and adjacent mesenchyme derived tissues (B. Zhang et al., 2016). In particular, the emergence of new dermal adipocytes depends on the establishment of a population of hair follicle TACs. Using a cell type-specific deletion of Smo, a gene required in SHH-receiving cells, Zhang et al. found that SHH released by TACs also acts directly on adipocyte precursors, promoting their proliferation and the expression of the Peroxisome proliferator-activated receptor *?* (PPARγ or PPARG), a key gene for the induction of dermal adipogenesis (Strzyz, 2016; Zhang et al., 2016).

Some lineage-tracing studies showed the existence in hair follicle of heterogeneous progenitors (i.e., TACs) that preferentially give rise to one or a number of the seven cell types present in the hair follicle (Legué et al., 2010). Using single-cell RNA-seq, Yang et al. traced the roots of this heterogeneity to micro-niches at the epithelial-mesenchymal interfaces, reflecting distinct local signals and intercellular interactions. By compartmentalizing SCs into micro-niches, a precise control over morphogenesis and regeneration is obtained. Some progenitors immediately give rise to specify lineages, whereas others maintain a self-renewing phenotype and only progressively restrict to specific lineages as they experience dynamic changes in microenvironment (Yang et al., 2017).

Dermal papilla cells from the postnatal skin retain the ability to direct epithelial cells. Almost 40 years ago Jahoda, Horne and Oliver were able to induce hair growth by implantation of the dermal cultured cells (Jahoda et al., 1984). Signal exchange between dermal papilla niche and SCs/TACs is still only partially understood. Formation of a new dermal papilla can be induced in adult skin by activating the Wnt pathway in the epidermis (Silva-Vargas et al., 2005). Interruption of beta-catenin signaling in the dermal papilla results in reduced cell proliferation of cells at the follicle base leading to a catagen stage and the prevention of anagen induction (Enshell-Seijffers et al., 2010). Beta-catenin controls several signaling pathways in dermal papilla cells which can act as mediators of the papilla inductive effects on the follicle epithelium. These signals include FGF signals such as Fgf7 and Fgf10 (Greco et al., 2009; Enshell-Seijffers et al., 2010). With next-generation RNA sequencing, Rezza et al. characterized transcriptomes of follicle and dermal papilla cells with the goal to define unique molecular signatures for SC precursors, TACs, and cell of the dermal papilla niche. They could show that hair follicle cells express a plethora of interesting ligands and receptors but were unable to obtain final conclusive information on markers for each cell differentiation stage (Rezza et al., 2016).

## SCs and TACs from different regions of the eye surface

Pellegrini et al. (1999a) isolated holoclones, meroclones, and paraclones from the entire human eye surface and found that bipotent holoclones could be obtained from the whole conjunctiva, in that they, in addition to conjunctiva keratinocytes, also generate goblet cells at least twice in their life. At variance with conjunctiva, holoclones forming corneal epithelium cells were strictly segregated in the limbus, a region at the border between the cornea and the sclera. Therefore, while in epidermis, hair follicles, and ocular conjunctiva TAC progeny are physically adjacent to SCs, TAC progeny of epithelial cornea can live and proliferate far from their SCs mothers. Corneal TACs (limbal SC progeny) migrate centripetally from limbus towards the central cornea to become corneal basal epithelial cells. This enables the investigation of SC properties *versus* their TAC progeny and terminally differentiated cells. Using a double-labeling technique that permits the detection of more than one round of DNA synthesis in a cell, Lehrer et al. showed the existence of a hierarchy in the cornea epithelial TAC population (Lehrer et al., 1998). While peripheral TACs undergo more than one replication cycle, those of the central cornea are capable of only one round of division before they become post-mitotic. Moreover, the cell cycle time of these central TACs is shortened and the number of replications is increased in response to a wound. Thus, three strategies of corneal epithelial repair exist: i) enhancing limbal stem cell replication to produce more TACs; ii) increasing the number of times a TAC can replicate; iii) increasing the efficiency of TAC replication via a shortening of the cycling time.

Single-cell RNA-sequencing (scRNA-seq) was used to identify SCs, TACs, and post-mitotic populations in limbal/corneal epithelia from mice. Kaplan et al. identified three limbal/corneal epithelial cell subpopulations designated as a combination of SCs and early TACs, mature TACs, and differentiated corneal epithelial cells (Kaplan et al., 2019). A second scRNA-seq study aimed at the characterization of the gene expression profile of a candidate TAC population in limbal basal epithelial cells was recently performed. This study assessed the status of early progenitors and status and enrichment of exclusive proliferation marker genes. In a cell population with 98.1% cells in S and G2/M phases, a cluster representing 3.2% of total cells was identified as a TAC entity. The cell cycle-dependent genes, largely enriched at both protein and mRNA level in this cell population, that could serve as TAC signature markers, were: RRM2, TK1, CENPF, NUSAP1, UBE2C, and CDC20 (J. M. Li et al., 2021).

## Control of SC and TAC balance in the epithelia

How the correct number of SCs, “early” and “late” TACs, and differentiated cells is maintained in a steady state self-renewing adult epithelial tissue is still not fully understood. Jensen and Watt generated cDNA libraries from single human epidermal cells, designated as SC or TAC and selected the EGF receptor antagonist leucine-rich repeats and immunoglobulin-like domains 1 (Lrig1) for further studies. Overexpression of Lrig1 decreased keratinocyte proliferation but did not affect the proportion of SCs and TACs. On the contrary, downregulation of Lrig1 stimulated keratinocyte proliferation in part by negatively regulating the Myc promoter. They proposed that Lrig1 maintains epidermal stem cells in a quiescent non dividing state, and that Lrig1 downregulation triggers proliferation (Jensen and Watt, 2006). At variance with the above, some authors proposed the existence of a single progenitor and considered quiescent cells as a reserve population to replace rapidly cycling stem cells periodically or after injury, their exact nature remaining unknown (Buczacki et al., 2013; Nusse and Klein, 2013; Zhu et al., 2013; Piedrafita et al., 2020). Indeed, the very methods used to identify intestinal stem cells may bias some of those results. Moreover, no specific markers have been so far identified to clearly distinguish between true SCs and multipotent early TACs.

Emerging evidence shows that SCs send feedforward signals to their progeny and that both negative and positive feedback and feedforward signals from SC progeny are likely to contribute to tissue homeostasis and regeneration (Tata and Rajagopal, 2016). In the mouse airway epithelium, trachea basal SCs and progenitor cells self-renew and differentiate to secretory and ciliated cells and to less represented, but disease-relevant cells, such as tuft, neuroendocrine, and ionocyte cells (Montoro et al., 2018). Secretory cells act as TACs that eventually give rise to post-mitotic ciliated cells (Pardo-Saganta et al., 2015). Pardo-Saganta and colleagues also showed that early progenitor cells continuously send a “feed-forward” Notch-based signal to the secretory cells thus preventing their uncontrolled differentiation to ciliated cells. This signal is essential for maintenance of the secretory daughter cells. Without these forward signals, the secretory progenitor cells execute a terminal differentiation program and convert into ciliated cells. Given that the Notch pathway regulates choice of secretory *versus* ciliated cell fate in both developing lung and regenerating adult airway epithelium (Tsao et al., 2009), Pardo-Saganta et al. assessed the expression of Notch pathway components in each cell type of the adult airway epithelium. Quantitative real time PCR analysis showed that the Notch1 receptor was highly expressed in basal stem and early progenitor cells, Notch2 and Notch3 were enriched in secretory progenitor cells, and Notch4 was not detected (Pardo-Saganta et al., 2015). As in epidermis, p63 plays a major role in the correct limbus development. In mice lacking p63, limbs are absent or truncated, defects that are caused by a failure of the apical ectodermal ridge to differentiate (Mills et al., 1999).

## The intestine crypt: a model to study SC, TAC, and post-mitotic cell interactions

Differentiated cells of the intestine lining, namely, absorptive, goblet, Paneth, and enteroendocrine cells, derive from a single type of stem cells. An additional cell population also derived from SCs are Tuft cells, i.e., taste-chemosensory cells, which monitor the intestinal content. Although very sparse in number, Tuft cells play a very important role in intestinal diseases and expand when parasites colonize or infect the gut (Hendel et al., 2022). Intestinal SCs lie at or near the base of crypts, finger-like invaginations of the epithelium into the underlying connective tissue. As SCs from other tissues, intestine SCs divide at a low rate and can either self-renew or give rise to a progeny. The daughter cells migrate upward from the depth of the crypts onto the surfaces of the villi, larger finger-like protrusions into the intestine lumen, taking up to 7 days to migrate from the crypt to their exfoliation point at the villus tip. The last cell cycle takes place at the crypt exit. In the villus no further cell division occurs, and all cells are fully differentiated. For the number of SCs in the crypt to remain stable, on the average, at each division a SC should generate a SC and a cell committed to differentiation. However, when Snippert et al. carried-out fate mapping of individual stem cells by generating a multicolor Cre-reporter, they observed that most SC divisions occurred symmetrically not supporting a model in which daughter cells from each SC division adopt divergent fates (i.e., one SC and one TAC) (Snippert et al., 2010; Buczacki et al., 2013). Given the continuous flow of cells from the crypts to the tips of villi and based on some experimental evidence it was suggested that intestinal SCs at the crypt bottom never enter a state of real quiescence. Instead, they divide at least once every 2 days and stochastically adopt SC or TAC fates (Lopez-Garcia et al., 2010; Snippert et al., 2010; Buczacki et al., 2013; Ritsma et al., 2014). By tracking individual stem cells over time, Ritsma et al. showed that the relative positioning of the cell within the niche stochastically regulates its fate (Ritsma et al., 2014). Stem cells located more close to the crypt base more likely persisted for long-term, whereas peripheral cells could more rarely move into privileged crypt-base positions.

**FIGURE 2:**
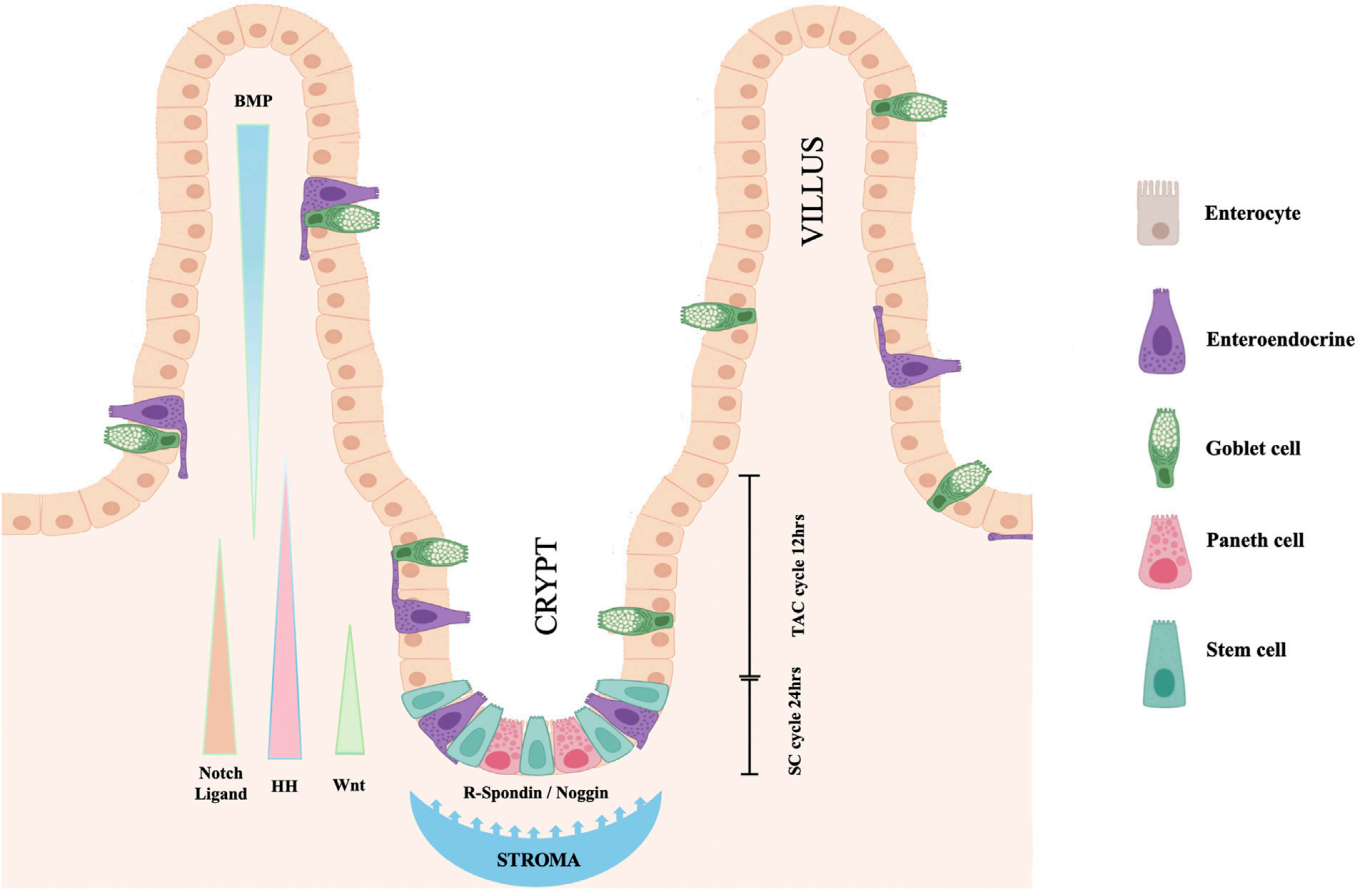
Cell turnover and proliferation in the intestinal epithelium. Resident SCs, dispersed at the crypt base among Paneth cells, double each day. Maintenance of the stem status depends upon the exposure of SCs to a Wnt signal released by Paneth cells. The Wnt signal also induces the crypt surrounding stroma to release R-spondin, Delta-ligand and other factors that stimulate SCs to exit quiescence. When SCs divide, they generate both new SCs and TACs. Notch-Delta signaling in combination with Wnt control proliferation and differentiation of TACs. Hedgehog signals are secreted by proliferating intestinal cells, stimulate surrounding mesenchyme to release BMPs and to block TAC proliferation favoring their differentiation. Nogging produced at the crypt base prevent the BMP action on the crypt cells allowing SC and TAC proliferation to continue (modification of figure templates created with BioRender.com).

Maintenance of the stem status depends upon the continuous exposure of SCs to a Wnt signal. Any interruption of this exposure stops SC division and movement away from the crypt base. Paneth cells located at the crypt base, themselves progeny of SCs, release EGF, TGF-alpha, Wnt3 and the Notch-ligand Delta (Dll4), all essential signals for stem-cell maintenance in culture (Figure 2). This Paneth cell requirement can be substituted by a pulse of exogenous Wnt (Sato et al., 2011). In response to the Wnt stimulus, also the connective tissue surrounding the crypt releases signaling molecules, R-spondin protein being one of the more relevant. A Wnt target gene is Lgr5 (leucine-rich-repeat-containing G-protein-coupled receptor 5, also known as Gpr49), the receptor for the R-spondin (Barker et al., 2007). Indeed, the crypt Lgr5 positive cells are true multipotent SCs with the potential to differentiate toward all four intestine cell phenotypes (Barker et al., 2007). A single Lgr5 positive cell can generate a complete, well-organized crypt-villus system (Sato and Clevers, 2013). R-spondin stimulates SCs to exit the quiescent state and proliferate. However, the Delta molecule released by nearby Paneth cells activates Notch in the SCs thus preventing their differentiation. At the same time, Notch-Delta signaling, in combination with Wnt, controls proliferation and differentiation of TACs. Wnt signals released by Paneth cells, and possibly by activated SCs, diffuse in the crypt and induces the adjacent TAC progeny to express both Notch and its ligand Delta. TACs expressing higher level of Delta can activate Notch in the neighboring TACs, but they stop dividing and differentiate to secretory cells. On the contrary, in the neighboring Delta/Notch activated TACs the differentiation is blocked, and, thanks to the presence of Wnt, the cells continue to proliferate and to migrate toward the villus. When they are out of the crypt and rich the villus, TACs escape from the Wnt influence, stop dividing and differentiate to absorptive cells. TACs regulate the release of R-spondin, and in turn the proliferation of SCs through the expression of URI. The protein encoded by this gene negatively modulates transcription through its binding to RNA polymerase II subunit 5 (RPB5). Genetic intestinal URI ablation in mice reduced the survival capacity of TACs and subsequently R-spondin levels, thereby causing SC quiescence and disruption of the intestinal structure (Chaves-Pérez et al., 2022).

In addition to R-spondin, the connective tissue surrounding the crypt and the intestinal epithelial cells interact and influence the cell fate through synthesis and release of other signaling molecules. For a review see (Crosnier et al., 2006). The identification and localization of pathways involved in determining the intestine cell fate has been most extensively studied in the mouse. In the mouse embryo, a major role for the development of the connective tissue surrounding the intestinal lumen is played by Hedgehog signals released by the intestinal epithelium (Apelqvist et al., 1997). In the adult intestine, SHH and IHH (Sonic and Indian Hedgehog respectively) are synthesized by the epithelium only where epithelial cell proliferation occurs, such as the intervillus region and the crypt bases, and diffuse outwards promoting villus formation and inhibiting new crypt formation near already existing crypts. This activates a feedback loop in which mesenchymal cells respond to the Hedgehog signals, sending in turn feedback signaling molecules to the epithelium. Signaling molecules delivered by mesenchymal cells to the epithelium include factors such as BMP-2 and BMP-4, two multifunctional growth factors, members of the Bone Morphogenetic Protein (BMP) family, belonging to the transforming growth factor *ß* (TGF-β) superfamily. Ligands of this family bind various TGF-beta receptors leading to recruitment and activation of SMAD family transcription factors (Miyazono et al., 2010). BMP signaling plays a major role in mediating the action of Hedgehog to block Wnt induced epithelial cell proliferation and the formation of ectopic crypts. Instead, Noggin, expressed near the crypt base, protects the epithelium in this region from the action of BMPs, and allows cell proliferation to continue and crypt to remain functional (Haramis et al., 2004). For recent reviews on Hedgehog and other regulatory signals in intestinal development and homeostasis see also (Kurokawa et al., 2021; Walton and Gumucio, 2021).

Thus far, despite the above information, molecular mechanisms and signaling pathways regulating and controlling SC proliferation and their differentiation to TACs are still not fully understood. Zhang et al. reported that loss of the Rho GTPase CDC42 in the intestinal SCs caused a drastic hyperproliferation of TACs and a disrupted epithelial polarity (Zhang et al., 2022). CDC42-null crypts presented an expanded TAC population and a diminished number of SCs. This was in part the result of an activation of the Hippo-Ereg-mTOR signaling cascade, independent from canonical Wnt signaling. In search of transcription factors whose expression was under the control of signaling molecules that modulate the transitions SCs to TACs and TACs to differentiated cells in the intestine, Xie et al. explored the role of the Zinc finger protein 277 (ZNF277), a classic C2H2 zinc finger transcription factor highly conserved in humans, and several animal species. They identified ZNF277 as a transcriptional target of beta-catenin signaling in the Wnt/β-catenin pathway and confirmed this by showing that beta-catenin knockdown reduced ZNF277 expression (Xie et al., 2022). In normal human small intestinal and colonic mucosa, ZNF277 protein expression was detected in undifferentiated rapidly cycling TACs, localized at the lower half of intestinal crypts, but not in differentiated enterocytes. They proposed ZNF277 as a potentially novel intestinal TAC marker.

## Gut micro-environmental changes due to inflammation, tissue injury or microorganism infection, interfere with the SC - TAC - post-mitotic cell transition

Inflammatory cytokines play an important role in SC/TAC regulation to initiate the immune response leading to tissue restoration and regeneration, especially after a tissue injury. IL-22, a cytokine of the IL-10 family immediately produced after insults such as inflammation, pathogen attack, and wounds, and Interleukin-10 itself promote intestinal TAC proliferation while depleting and reducing Lgr5 cell survival by inhibition of Notch and Wnt signaling (Zha et al., 2019; Deng et al., 2020). Zwarycz et al. showed that in an *in vitro* ileal organoid model, the stimulation with IL-22 increased the organoid size, but decreased the organoid survival, reducing expression of SCs markers (Lgr5, Olmf4) and Wnt and Notch signaling (Zwarycz et al., 2019). Another class of pro-inflammatory cytokines influencing crypt cell proliferation and differentiation are the members of the IL-6 family. IL-6 normally activates JAK–STAT3 signaling *via* the co-receptor gp130. However, in mice and human injured intestinal tissues, gp130 also triggers activation of YAP and Notch, transcriptional regulators of tissue growth and regeneration, independently of the gp130 effector STAT3 (Taniguchi et al., 2015). Nevertheless, autocrine IL-6 signaling in the gut epithelium regulates crypt homeostasis through the Paneth cells and the Wnt signaling pathway. Using mouse *in vivo* models and *in vitro* crypt organoid, Jeffery et al. showed that exogenous IL-6 promoted crypt organoid proliferation and increased stem cell numbers through pSTAT3 activation in Paneth cells (Jeffery et al., 2017). They also indicated that the IL-6 receptor was localized at the basal membrane of Paneth cells and that the crypt epithelium expressed IL-6. Interestingly, Interferons (IFN), signaling proteins mainly released by cells in response to the presence of viruses, are also important inducers of the regenerative capacity of intestinal SCs. Interferon signaling preserves SC stemness by restricting secretory-cell differentiation (Sato et al., 2020).

To measure intestinal epithelial cell-type composition changes in response to microenviron-mental signals, Sanman et al. utilized an enteroid monolayer culture system that maintains characteristics of intestinal epithelial architecture, including organization into crypt-like proliferative and differentiated compartments and apical-basolateral polarization (Sanman et al., 2021). When they investigated the effects of combinatorial signaling perturbations on intestinal cell fate, they found an unexpected and unexplained mutual antagonism between IL-4 and Epidermal growth factor receptor-inhibitor (EGFR-i) in the control of TAC numbers. The presence of IL-4 at a tissue injury site inhibited classical activation of macrophages into pro-inflammatory M1 cells and promoted alternative activation of macrophages into pro-resolving M2 cells. EGFR inhibitors, like gefitinib and other Tirosine Kinase Inhibitors (TKIs) blocked induction of the receptor intracellular tyrosine kinase-mediated signaling pathways. In the enteroid monolayer culture, IL-4 or EGFR-i individually reduced the number of TACs relative to control by about 4 or 6 log-fold respectively, while co-treatment reduced the TAC number by only 1.5 log-fold. The modulation of TAC proliferation changed the ratio of differentiated secretory to absorptive cell types highlighting a role for TACs in tuning intestinal differentiated cell-type composition (Sanman et al., 2021).

## Mesenchymal Stem Cells (MSCs) and transit amplifying Cells (TACs)

Even though to date most studies focused on tissues and organs of ectodermal origin, colony-forming cells of mesodermal origin, subsequently named Mesenchymal Stem Cells (MSCs), were first isolated in the late sixties from bone marrow (Friedenstein et al., 1968; Kuznetsov et al., 1997). Since then, MSCs with similar properties, including multipotent differentiation potential, have been isolated from several other tissues, such as adipose, skeletal muscle, bone, placenta, etc. The definition of MSCs is based on loose criteria including trilineage (bone, adipose, cartilage) *in vitro* differentiation, the expression of some specific surface markers and the absence of hematopoietic markers. However, the classification of MSCs as a homogeneous, true stem cell population is very questionable since MSCs are a mixture of possibly rare SCs and a large majority of SC progeny cells at different degree of differentiation. They should be more appropriately considered mesenchymal stromal cells (Keating, 2012). Moreover, at variance with epithelial SC and with the exclusion of the hematopoietic system, the *in vivo* supporting niche of SCs in tissues of mesodermal origin and how these SCs interact with their TAC progeny to maintain tissue homeostasis remains essentially unknown.

Muraglia et al. (2000) showed by limiting cell dilution that bone-marrow MSC clones derived from single cells presented a multilineage differentiation potential and a self-renewal capacity. These authors proposed a hierarchical model in which there was sequential loss of lineage potential from the initial osteo-chondro-adipogenic to osteo-chondrogenic, and eventually to osteogenic precursors. Osteo-adipogenic and chondro-adipogenic precusors were not detected, nor were only chondrogenic or adipogenic clones. Notably, MSCs were derived from bone-marrow and cells were cultured in culture conditions favoring osteogenic differentiation, i.e., on plastic Petri dishes in the presence of 10% fetal calf serum and in the absence of any supplemented specific growth factor inducer. Similar results were obtained by Lee et al. which conducted single-cell studies of GFP-marked human MSCs and showed that a minor subpopulation exhibited differentiation along osteogenic, chondrogenic, and adipogenic lineages and could self-renew from colony replating assays (Lee et al., 2010). However, when bone-marrow MSCs were cultured in high-density “pellet culture”, they underwent chondrogenesis and formed a tissue that was morphologically and biochemically defined as cartilage (Muraglia et al., 2003). The clonogenic differentiation capacity of human umbilical cord MSCs, was investigated by Surugaser et al. They reported the existence of a rare self-renewing MSC population able to differentiate to myogenic, osteogenic, chondrogenic, adipogenic, and fibroblastic lineages and proposed a hierarchical stem cell lineage relationship for these cells (Sarugaser et al., 2009).

The balance between the more immature stem/progenitor cells and the already committed progenitor/post-mitotic cells is significantly dependent on the culture microenvironment including nature and morphology of substrates and composition of the culture medium. Properties of the substrates, such as 2D *versus* 3D scaffolds, microstructure (including fiber diameter, pore size, and scaffold alignment), physicochemical (elasticity, stiffness, etc.) and biochemical properties drive cell behaviors including migration, proliferation, and differentiation. (Kennedy et al., 2017). The degree of matrix deformation has implications for intracellular mechano-signaling, leading to distinct differentiation pathways in MSCs. When substrates with similar surface chemistry and varying stiffnesses and topographies were prepared, and Wharton’s Jelly Umbilical Cord Mesenchymal Stem Cells were cultured on these substrates, it was observed that soft substates improved cell multiplication and migration, while stiff substrates induced differentiation of MSCs into bone cells (Kozaniti et al., 2022). In another study, the role of stiffness was examined in MSC differentiation to two closely related cell phenotypes: osteoblast and chondrocyte. Four methyl acrylate/methyl methacrylate (MA/MMA) polymer surfaces with different elastic moduli were prepared. MSCs on lower stiffness substrates showed elevated expression of cartilage markers, whereas on substrates with increased stiffness the expression of osteoblasts specific markers was higher (Navarrete et al., 2017). In line with these findings, although alumina ceramics are bioinert, alumina-coated implant surface promoted MSC commitment to the osteoblast phenotype also in the absence of specific induction (Mendonça et al., 2009). Higher levels of osteoblastic differentiation markers such as alkaline phosphatase, osteocalcin, and mineralization were detected in cells cultured on alumina with 100 nm pores or wider groves (100 μm/50 μm groove/pitch) compared with cells cultured on alumina with either 20 nm pores or narrow groves (10 μm/10 μm groove/pitch) or smooth alumina (Nadeem et al., 2013; Song et al., 2013). Indeed, in the MSC population derived from bone marrow and expanded *in vitro* in standard plastic Petri culture dish, the colony forming units of the osteogenic lineage (CFU-OBs) have the characteristics of TACs, i.e., cells capable of a limited number of self-renewal cell divisions, eventually giving rise to differentiated cells. In the same culture condition, supplementation of FGF-2 in the culture medium promoted a more extensive proliferation of human more immature MSCs but maintaining the cell osteogenic potential. When implanted *in vivo* after seeding on ceramics, these cells differentiated, and formed an ectopic bone tissue in immune-compromised mice (Martin et al., 1997). In a similar *in vivo* bone formation model, an intrinsic capability of mouse bone-marrow MSCs to activate endogenous regenerative mechanisms in the host without a direct bone deposition by the implanted cells, was shown to be critically dependent on the MSC commitment level. The presence of FGF-2 in the culture medium during mouse MSC expansion *in vitro* was the key factor. Only MSCs expanded in the presence of FGF-2, and not MSCs expanded in the absence of the factor, induced a host regenerative response *in vivo* leading to an endochondral bone formation by the cells of the host itself (Tasso et al., 2012).

## How the MSC lineage commitment is controlled?

For adipogenic differentiation, MSCs are usually cultured in medium supplemented with isobutylmethylxanthine or indomethacin, dexamethasone, and insulin. For osteogenic differentiation, MSCs are usually cultured in medium containing Dex, L-ascorbic acid, and beta-glycerophosphate. However, when the progression of MSCs to post-mitotic differentiated cells was more carefully investigated, it was observed that this is a two-step process: lineage commitment (from uncommitted TACs to lineage specific TACs) and maturation (from progenitors to specific cell types). Bone-marrow stromal cells can differentiate into multiple mesenchymal lineages including cartilage and bone. In conventional culture conditions the preferred lineage is the osteogenic. When the cells are cultured in high-density ‘pellet culture’, they undergo chondrogenesis and form a tissue morphologically and biochemically identical to cartilage (Muraglia et al., 2003). Starting from the cloning work of Muraglia et al. an increased body of evidence indicates that adipogenesis and osteogenesis of MSCs are competing and reciprocal processes. Several biological, chemical, and physical inputs act through a variety of signaling pathways to favor adipogenesis and osteogenesis respectively.

The TGFβ/BMPs signaling pathway has been generally recognized to have dual roles in regulating MSC adipogenic and osteogenic differentiation (Kang et al., 2009). Increasing evidence suggests an important role of Wnt signaling (Li et al., 2017; Yang et al., 2016). Wnt activation favors osteogenic differentiation (Park et al., 2015) and inhibits adipogenic differentiation of MSCs (Yuan et al., 2016). Hedgehog signaling pathway is pro-osteogenic and anti-adipogenic (Spinella-Jaegle et al., 2001; James et al., 2010). Notch signaling pathway regulates both adipogenesis and osteogenesis of MSCs through direct target genes or interacting with other signaling pathways (Lin and Hankenson, 2011; Song et al., 2015). Additional signaling pathways, including FGFs, PDGF, EGF, and IGF were shown to regulate adipogenic and osteogenic MSC differentiation, in several cases through the involvement of other pathways, such as Wnt and TGFβ/BMP. For a dedicated review see (Chen et al., 2016). Increasing evidence suggests that also miRNAs have roles both in lineage commitment of MSCs and for cell terminal differentiation. Some miRNAs have been identified as regulators of osteogenic differentiation, including miR-125b (Mizuno et al., 2008), miR-26a (Luzi et al., 2008), miR-196a (Kim et al., 2009), miR-194 (Jeong et al., 2014), miR-204/211 (Huang et al., 2010), miR-149 (Fan et al., 2022), and miRs-148b, −27a and −489 (Schoolmeesters et al., 2009). Other miRnas such asMiR-637 and miR-27b maintain the balance between adipocytes and osteoblasts and control adipocyte differentiation (Karbiener et al., 2009; Zhang et al., 2011). miRNAs are also found in exosomes, small extracellular vesicles rich in molecular cargos, secreted by cells for intercellular communication. Recently, many studies have focused on miRNAs released by cells within exosomes as promising therapeutic factors to support tissue regeneration and to treat tumors. In several cases exosomes loaded with miRNAs were from MSCs (Foo et al., 2021). However, specific targets for most miRNAs are not yet fully identified. It has been reported that miR-335 orchestrates cell proliferation, migration, and differentiation in human MSCs (Tomé et al., 2011). However, the role of miRNAs in MSC activities, such as cell migration and proliferation, is still essentially unknown.

Eventually, all these signals converge at a cascade of transcription events, including C/EBPs and PPARγ for adipogenesis and Runx2 and Osterix for osteogenesis. C/EBPs (CCAAT-enhancer-binding proteins) are a family of transcription factors including six members. Two of these transcription factors, C/EBP-beta and C/EBP-delta, are transiently expressed during the early stages of adipogenesis and concur to the induction of the expression of the “masters” adipogenic transcription factors C/EBP-alpha and PPAR-gamma. In fact, C/EBP-alpha is upregulated at a later adipogenesis stage and possibly promotes adipogenesis by inducing the expression of PPARγ. Regarding the osteogenesis control, during osteoblast differentiation, Runx2 is crucial for the commitment of mesenchymal stem cells to the osteoblast lineage and positively influences early stages of osteoblast differentiation. In osteoblast biology, RUNX2 regulates the process of osteoblast differentiation at different stages. Regulation by RUNX2 takes place in a positive manner at early stages of differentiation, while RUNX2 inhibits the process at later stages (Bruderer et al., 2014). Osterix (OSX) starts playing an important role in osteoblast differentiation following Runx2-mediated mesenchymal condensation. Osx affects differentiation, maturation, and function of bone cells. It is expressed in both osteoblasts and osteocytes and decreases osteoblast activity by stimulating the expression of genes predominantly expressed in osteocytes, such as sclerostin (Sost) and Wnt signaling pathway inhibitors. Moreover, Osx represses adipogenesis by negatively regulating PPAR-gamma expression (Liu et al., 2020) and regulates osteogenesis of human MSCs (Onizuka et al., 2016). Additional relevant players in the progression of MSCs to post-mitotic differentiated cells are YAP/TAZ signaling components, intracellular messengers communicating extracellular biophysical and biochemical stimuli to the nuclear transcription apparatus and back to the cell/tissue microenvironment interface through the regulation of cytoskeletal and extracellular matrix components (Kovar et al., 2020). YAP stabilizes nuclear beta-catenin, while TAZ binds to SMAD4 co-activating RUNX2 to drive osteoblast differentiation of MSCs and inhibit gene transcription induced by adipogenic peroxisome proliferator-activated receptor gamma (PPAR-gamma) (Lorthongpanich et al., 2019; Pan et al., 2018; Park et al., 2019). Several microRNAs are involved in the control of YAP/TAZ activity. For example, miR-135b-5p and miR-33-5p and -3p were implicated in osteogenic priming of MSC through indirect control of YAP and TAZ expression and nuclear translocation (Si et al., 2017; Costa et al., 2019).

Moreover, a control on MSC differentiation, especially *in vivo*, is also exerted by hormones. Consistent with a direct receptor-mediated action of estrogens on early mesenchymal cell progenitors, estrogens reduced by about 50% the self-renewal of mouse bone-marrow CFU-OBs (i.e., TACs already osteogenic committed). Given this result and in agreement with evidence that osteoblasts are required for bone deposition, but also for osteoclast development, it was suggested that this may represent a key mechanism also for the *in vivo* anti-bone remodeling effects of estrogens (di Gregorio et al., 2001).

## The mouse incisor: a model to investigate epithelial—mesenchymal cell interactions during tooth regeneration

So far, for SC and TAC of mesodermal origin, a deeply investigated model, such as the intestinal crypt for epithelial SC and TAC, does not exist. Some authors have proposed the mouse incisor to study reciprocal paracrine interactions between SC and TAC during the tooth regeneration. In fact, at variance with most mammals, in the mouse the incisor grows throughout the whole animal life. The mouse incisor presents an external layer of enamel deposed by dentin odontoblasts at the periphery and an inner dental pulp that contains vessels and nervous tissue. The epithelial odontoblasts and the mesenchymal cells of the dental pulp renew all their cells about every month. Zhao et al. identified the neurovascular bundle as an MSC niche. They found that sensory nerves secrete Shh protein, which activates Gli1 (a transcriptional effector at the terminal end of the Hedgehog signaling pathway) expression in quiescent periarterial cells. Dental pulp cells expressing Gli1 have properties of mesenchymal SCs continuously dividing and these cells, located at the incisor proximal end, generate TACs in the immediately adjacent region (Zhao et al., 2014). These TACs proliferate, and give rise to committed pre-odontoblasts, post-mitotic differentiated odontoblasts, and dental pulp cells. An et al. identified PRC1 (Polycomb Repressive Complex 1) as a controller of TACs *via* WNT/beta-catenin signaling and reported that the TAC population unable to self-renew is characterized by the expression of Axin 2, an inhibitor of the canonical Wnt signaling pathway in form of a negative feedback loop (An et al., 2018). More recently the reciprocal interactions between mesenchymal SCs and TACs that regulate mouse incisor homeostasis were more extensively investigated by Jing et al. Significant findings were: i) MSCs feedforward to TACs through an IGF-WNT signaling cascade; ii) the control of TAC fate depends on tissue-autonomous canonical WNT signaling; iii) TACs produce Wnt5a, which provides feedback to mesenchymal SCs via the beta-catenin antagonizer Ror2-mediated non-canonical WNT signaling (Jing et al., 2021). Epigenetic modifications may also play a role. Arid1a, a core component of the SWI/SNF complex (SWItch/Sucrose Non-Fermentable complex, a group of proteins that associate to remodel the way DNA is packaged) performs epigenetic regulation of stage-specific and tissue-specific genes that are indispensable for mouse incisor SC homeostasis and differentiation, although the functional mechanism is not clear (Du et al., 2021). Arid1a also limits proliferation and promotes cell cycle exit and differentiation of TACs by inhibiting the Aurka-Cdk1 axis. Loss of ARID1A expression results in an enhanced AURKA transcription, which leads to the persistent activation of CDC25C, a key protein for G2/M transition and mitotic entry thus leading to expansion of the mitotic TAC population but compromising their differentiation ability.

Given the specificity of the mouse incisor model, it remains to be demonstrated if the mesenchymal SC-TAC interaction mode and the control mechanisms described for this tooth could potentially apply to other organs where existing SC-TAC interactions are essentially unknown.

## Dedifferentiated cells: a reversion from post-mitotic to transit amplifying cells

Dedifferentiation is a process by which terminally differentiated cells revert to an earlier differentiation stage within their own lineage. Mainly based on knowledge about blood cell generation starting from hematopoietic SCs, for long time the differentiation from SCs to post-mitotic cells was considered a one-way irreversible process. Today, increasing evidence suggests that in special circumstances, already differentiated cells can revert to a pre-differentiation stage. Dedifferentiation/re-differentiation is a process relatively frequent in plants. In vertebrates a cell re-programming capacity is present during embryogenesis but, after birth, this capacity remains only in some lower vertebrate animals. Classical vertebrate models where post-mitotic cell dedifferentiation up to a stem stage and subsequent redifferentiation of these cells has been investigated, are salamanders and zebrafish. By a cell dedifferentiation/redifferentiation process, adult newts can regenerate limbs, tail, jaws, spinal cord, retinas, lenses, optic nerves, intestine, and part of the heart ventricle (Odelberg, 2005; Tanaka, 2016). In the zebrafish, resection of up to a fifth of the heart ventricle regenerates through the proliferation of only a few differentiated cardiomyocytes (Jopling et al., 2010; Kikuchi et al., 2010). Interestingly, limb regeneration occurs in tadpoles, but not in limbs of frogs despite the formation of a blastema with several characteristics like the ones of newt and axolotl. There are two possible reasons behind the frog blastema behavior: an intrinsic incapability of cells to form competent stem cells or a non-permissive environment in the adult frog compared to the embryo environment. To understand what blastema cells from adult frog can do when decoupled from their environment, blastema cells were transplanted in the developing limb bud of stage 50 tadpoles (15 days p. f.), a microenvironment permissive to complete limb development. Frog blastema cells did not fully re-express the limb bud progenitor program revealing their intrinsic inability to be reprogrammed back to a stem cell stage (Lin et al., 2021). More recently, extensive research has been done on the *in vivo* cell dedifferentiation and reprogramming occurring during wound repair in mammals. At variance with invertebrate animals that harbors pluripotent SCs undergoing forward differentiation to regenerate even after a body large amputation (Reddien, 2013), only few mammalian tissues with a physiological high cell turnover, such as skin, intestine, and the blood system, undergo a regeneration process dependent on the existence of tissue resident SCs. Interesting, in the tissues where SCs are present and drive the tissue regeneration/repair, SCs also prevent dedifferentiation of already differentiated cells to occur possibly via paracrine signals. Interestingly, some authors reported that in the mouse intestine, after an injury, enterocytes and Paneth cells can dedifferentiate to give rise to cells with stem properties able to persist in the intestine (Tetteh et al., 2016; Yu et al., 2018). This should maintain the correct balance between SCs and their progeny. Most mammalian tissues have a very limited regenerative capacity, and their ability to regenerate after an injury is dependent on the reversion of already differentiated cells to progenitor cells, their possible proliferation and their final redifferentiation (Pesaresi et al., 2019). There is some support for the presence in already differentiated cells of a program to regain regenerative ability and the possible activation of the program in certain circumstances (Messal et al., 2018). This has been shown especially in pancreas, liver, and kidney. Apparently, the reverted progenitor stage in most cases correspond to early or late TACs and not to SCs and their redifferentiation results in the formation of the original differentiated cell type. It should be better defined if this is due to the maintenance by the cells of a memory of the initial lineage or to the exposure of the cells to the same microenvironment than before the injury, or both.

Therefore, dedifferentiation and reprogramming seems to be a general property of post-mitotic differentiated cells after a tissue injury. For additional information see two recent reviews dedicated to dedifferentiation (Yao and Wang, 2020; Yanjie et al., 2022). However, this only occurs after modifications of the environment surrounding the cell, tissue damage being one of the main causes of these modifications. Environmental changes expose cells to new stimuli that either directly promote dedifferentiation and reprogramming or relieve inhibitory signals that block any phenotypic changes and are the driving factors for the dedifferentiation and subsequent reprogramming of the wounded tissue cells. At the wound site, blood flow is interrupted due to vascular injury and a local tissue hypoxia is generated, where hypoxia-inducible factor (HIF) become stabilized (Ruthenborg et al., 2014). It is known that hypoxic conditions promote self-renewal and pluripotency maintenance in embryonic and other SCs (Danet et al., 2003; Yoshida et al., 2009; Arthur et al., 2019). Hypoxia is also known to favor the expression of pluripotent factors, such as OCT3, OCT4, SOX2, NANOG and Krüppel-like factor 4 (KLF4) (Yoshida et al., 2009). Moreover, under hypoxia, most eukaryotic cells switch from mitochondrial respiration to glycolysis to maintain ATP levels. Previous studies have shown that high levels of glycolysis can maintain the self-renewal properties of stem cells (Kondoh et al., 2007; Simsek et al., 2010; Suda et al., 2011). The metabolic conversion from oxidative phosphorylation to aerobic glycolysis plays a key role in cell dedifferentiation/regeneration.

Another consequence of blood vessel injury is that platelets become activated when exposed to extravascular collagen and release their content of soluble mediators. After the initial hemostasis, wound healing progresses through additional partially overlapping phases: inflammation and tissue remodeling. Platelet released molecules and a transitory level of inflammation appears to be critical for tissue repair (R. Cancedda et al., 2017). Temporally regulated activation and suppression of inflammation was shown to play a role for achieving effective cardiac repair and regeneration (Jiang and Liao, 2010; Cooke, 2019). Studies on nuclear reprogramming have indicated that the generation of iPSCs from somatic cells requires the activation of innate immunity in addition to the forced overexpression of pluripotency genes and the suppression of genes enforcing the somatic cell lineage (Cooke et al., 2014; Lee et al., 2012). Indeed, acute inflammation triggers tissue repair or regeneration. Instead, within a chronic inflammatory response, the cells are not effectively reprogrammed, and tissue regeneration is impaired (Pietras et al., 2016). Dedifferentiation and reprogramming could still occur during chronic inflammation, but this may be one cause of carcinogenesis (Ben-Neriah and Karin, 2011).

Platelets contains a cocktail of growth factors and cytokines, which actively triggers innate immune cell migration (granulocytes and monocytes) to the wound. Together with factors secreted by migrated immune cells, platelet factors create an inflammatory microenvironment, in turn, causing angiogenesis and vasculogenesis activation. Eventually, regeneration or repair of the injured tissue occurs *via* paracrine signals activating resident SCs when they are present in the wounded tissue or inducing dedifferentiation and reprogramming of the tissue differentiated cells. In this regard, platelet lysate (PL) can act directly on the cells at the wound site or indirectly through an enhancement of the inflammatory response. In quiescent human osteoblasts, PL stimulation induced a transient increase of NF-kB activation, COX-2 induction, and secretion of pro-inflammatory cytokines (Ruggiu et al., 2013). In an *in vitro* scratch assay, PL promoted the wound closure by human keratinocyte proliferation and migration. This was associated with a high expression of the inflammatory cytokine interleukin-8 and an activation of the inflammatory pathways, p38 protein kinase, and NF-κB, a transcription factor typically activated during inflammation (Backly et al., 2011). At a later stage, PL in the inflammatory milieu exerted a protective effect on human umbilical vein endothelial cells **(**HUVEC**)** by inhibiting IL-1α-activated NF-κB pathway and by inducing the secretion of PGE_2_, a pro-resolving molecule in the wound microenvironment (van Nguyen et al., 2018). Moreover, PL enhanced HUVEC proliferation, without affecting their differentiation capability, and activated resting quiescent cells to re-enter the cell cycle. In agreement with these findings, proliferation-related pathways Akt and ERK_1/2_ were activated. The expression of the cell-cycle activator Cyclin D1 was also enhanced, as well as the expression of the High Mobility Group Box-1 (HMGB1), a protein of the alarmin group involved in tissue homeostasis, repair, and remodeling (van Nguyen et al., 2018).

However, despite the large number of articles dealing with molecular mechanisms controlling regeneration in amphibia after limb amputation, up to now, little work has been done on molecular mechanisms that control post mitotic cell reversion to a progenitor stage in mammals. Genes and regulatory pathways involved in the dedifferentiation process are not fully understood and are still being investigated. Several pathways known to play a role during SC differentiation are playing a role also in the dedifferentiation process. The BMP pathway is necessary for dedifferentiation and regeneration in tadpoles (Beck et al., 2003). The Notch pathway is important in regeneration of frog tadpole tails. Lowered Notch expression resulted in no tadpole tail regeneration, and induced Notch expression could partially rescue tail regeneration (Beck et al., 2003). The tumor suppressor retinoblastoma protein (pRB) maintains G0 arrested SCs in a quiescent state. Inactivation of pRb leads SCs to exit from quiescence and to increase their cell number without loss of their self-renewal capacity. In cycling progenitors, pRb plays a role at the G1, S, and G2 checkpoints and promotes cell differentiation (Burkhart and Sage, 2008). A transient combined inactivation of both pRB and ARF (p14 from Alternate Reading Frame) has been reported to promote dedifferentiation and proliferation of mammalian muscle cells (Pajcini et al., 2010). When in conjunction with Nanog, the canonical Wnt pathway induced partial dedifferentiation in zebrafish endothelial cells (Kohler et al., 2014). NF-κB modulates Wnt/beta-catenin signaling and its inactivation retards crypt SC expansion. On the contrary, elevated NF-κB signaling enhances Wnt activation and induces dedifferentiation of non-stem intestinal cells (Schwitalla et al., 2013). In the pancreas, NF-κB can trigger the beta-cells and acinar cells dedifferentiation (Murtaugh and Keefe, 2015).

To allow dedifferentiation, in addition to changes in the autocrine and paracrine signaling, a rise in proteolytic activities contributes to modify the cell microenvironment by freeing the cells from their interaction with the extracellular matrix. These proteolytic activities include Matrix Metalloproteinases (MMPs), responsible for degradation of both non-matrix and matrix proteins, that are upregulated also during early stages of limb regeneration (Chaar and Tsilfidis, 2006; Nagase et al., 2006).

## *In vitro* cultured cells: are they equivalent to transit amplifying cells?

The establishment *in vitro* of a primary cell culture, following a proteolytic treatment of the tissues from which they derive, is essentially equivalent to the occurrence of an *in vivo* tissue wound. Not only there is a loss of the interactions of the cell surface proteins with the surrounding extracellular matrix, but also the culture conditions are a dramatic change compared to the paracrine milieu the cells had *in vivo*. In general, especially for tissues of mesodermal origin, cultured cells are characterized by changes in shape, and in patterns of gene and protein expression compared to morphology and gene and protein expression of cells of tissues by which they derive. Moreover, they revert to a proliferative state, and acquire properties of *bona fide* TACs. As TACs, although they may preferentially re-differentiate into the same original lineage, in several cases, they may differentiate to cell types other than they were prior to dedifferentiation. The investigation of mechanisms of *in vitro* cell dedifferentiation and re-differentiation and of the nature of cultured dedifferentiated cells has not only an academic interest to advance our knowledge but is highly relevant to the possibility of adopting these cells for new regenerative medicine treatments. In recent decades, an increasing number of terminally differentiated cells obtained in a non-invasive manner were propagated *in vitro* as reverted progenitors or dedifferentiated cells and transplanted *in vivo* to achieve regeneration of damaged tissues and organs.

Examples of cultured cells with TAC characteristics adopted to repair damaged epithelia are cultured keratinocytes from epidermis (Gallico et al., 1984; de Luca et al., 1989), urethra (Romagnoli et al., 1990; Raya-Rivera et al., 2011), corneal epithelium (Pellegrini et al., 1997; Guérin et al., 2022). We already discussed at length in a previous section of this review why cultured Mesenchymal Stem Cells (MSCs), derived in most cases from bone-marrow or adipose, could be regarded as TACs. MSCs have a limited cell proliferation and are not immortal as true embryonic SC and cancer cell lines are. MSCs preferentially re-differentiate into the original lineage but, as for early TACs, by changing the culture environment it is possible to redirect their differentiation toward alternative lineages. MSCs through the secretion of factors and the release of extracellular vesicles can exert a paracrine control on other cell types. The adoption of *in vitro* expanded MSCs in regenerative medicine was first reported in 2001 where large bone defects in three patients were treated with ceramic scaffolds seeded with autologous MSCs (Quarto et al., 2001). Since then, during the last more than 20 years, MSCs became the more implanted cultured cells for the treatment of different pathologies. Human clinical trials were proposed taking advantage of either the direct transformation of the implanted MSCs into cells of the receiving tissue or the MSC immunomodulatory and paracrine induction exerted on tissue resident cells. At the time this review is compiled, during the last 5 years, 855 clinical trials matching mesenchymal stem cells in the title abstract have been registered in the Cochrane library, a collection of databases to make the results of controlled trials readily available (https://www.cochranelibrary.com).

The chondrocyte dedifferentiation and redifferentiation is another example of a cell culture system well documented and deeply investigated. In this review we will describe and report literature on the chondrocyte culture with the idea that this could be considered a paradigm for other cell systems and that the acquired information could be extrapolated to culture, dedifferentiation, and re-differentiation of other cell types.

In the 80s and the first half of the 90s of last century the fate of cultured chondrocytes was a major research topic for several teams of cell biologists. The differentiated phenotype of rabbit articular chondrocytes consists primarily of type II collagen and cartilage-specific proteoglycan. During serial monolayer culture the rabbit articular chondrocytes acquired a flattened anchorage-dependent and changed secretory phenotype from the synthesis of type II collagen and a high level of proteoglycan to the synthesis of type I collagen and of a low level of proteoglycan. However, when transferred to 0.5% low Tm agarose gel, an anchorage-independent culture, dedifferentiated chondrocytes reacquired the spherical morphology and re-express the differentiated phenotype. The rates of proteoglycan and type II collagen synthesis returned to those of primary chondrocytes (Benya and Shaffer, 1982).

**FIGURE 3:**
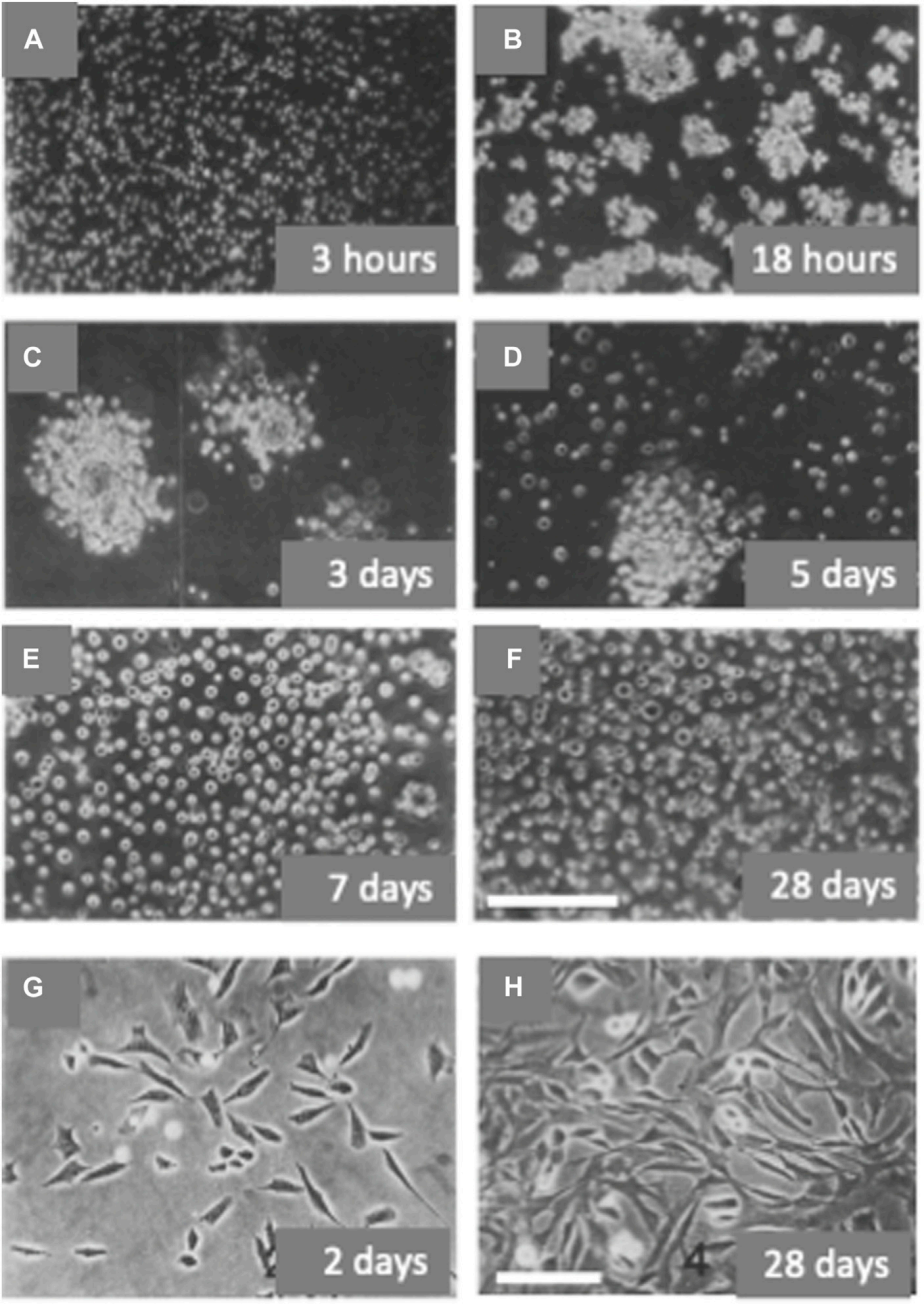
*In vitro* differentiation and de-differentiation of avian chondrocytes. Chondrocytes obtained from the digestion of tibiae of stage 29–31 chick embryos were cultured in anchorage non-permissive dishes and passaged weekly by direct dilution in fresh medium without prior harvesting by centrifugation [Panels (**A–F**)]. Chondrocytes were also plated at the same density in anchorage permissive dishes and passaged weekly 1:3/1:4 after trypsin digestion [Panels **(G–H)]**. Cells were observed by phase-contrast microscopy at different time intervals after the beginning of the culture. Bar: 200 microns **(A–F)**; 100 microns **(G–H)** [Modified from (Castagnola et al., 1986)].

In the developing chick embryo tibia, type II collagen is synthesized by chondrocytes in the resting and proliferative zones and type X collagen is synthesized only by chondrocytes from regions of hypertrophy (Capasso et al., 1984; Schmid and Linsenmayer, 1985). Freshly dissociated chondrocytes from 29–31-stage chick embryo tibiae are small and synthesize type II and not type X collagen. When cultured in non-permissive anchorage conditions these chondrocytes within few hours formed “organoid like” aggregates. From the second to the fifth day of culture the cells in the aggregates progressively increased in size and number and, at the same time, they started to “flourish”, decreasing their compactness, and releasing hypertrophic chondrocytes as isolated cells (Figures 3A–F). By the seventh day the culture was formed mostly by hypertrophic chondrocytes synthesizing type X collagen. The synthesis of type X collagen was monitored in cultured cells by analysis of labeled collagens and *in vitro* translation of mRNAs (Castagnola et al., 1986). Instead, when the freshly dissociated tibial chondrocytes were plated in anchorage-permissive dishes, most cells attached and dedifferentiated, assuming a fibroblastic morphology and synthesizing type I collagen (Figures 3G, H). If returned to a non-permissive anchorage culture, dedifferentiated chondrocytes could re-express the differentiated phenotype and continue their development to hypertrophic, type X collagen-synthesizing chondrocytes (Castagnola et al., 1986). Hypertrophic chondrocytes, grown in suspension culture to the stage of single cells as described above, were transferred to substrate-dependent culture conditions in the presence of ascorbic acid. Cells showed a change in morphology, became more elongated and flattened, expressed alkaline phosphatase, and eventually deposited a bone-like mineralized matrix. Type II and X collagen synthesis was halted and replaced by type I collagen synthesis (Cancedda et al., 1992). The cell cycle kinetic characteristics of chick endochondral chondrocytes differentiating *in vitro* were studied by flow cytometry. Dedifferentiated chondrocytes synthetizing type I collagen had a total cell cycle time of about 17 h (tG1 = 8 h, tS = 5 h, and tG2 + M = 4 h). In cultures of hypertrophic chondrocytes characterized by type X collagen synthesis, a low growth fraction (GF = 0.52) was observed with a total cell cycle time of the proliferating cells of about 73 h (tG1 = 53 h, tS = 12 h, and tG2 + M = 8 h) (Giaretti et al., 1988).

Gerstenfeld et al. defined culture conditions for promoting growth, hypertrophy, and extracellular matrix mineralization of embryonic chick vertebral chondrocytes. Ascorbic acid supplementation by itself led to the hypertrophic phenotype and type X synthesis. Maximal extracellular matrix mineralization was obtained when cultures were grown in medium supplemented with both ascorbic acid and 20 mM beta-glycerophosphate. Ultrastructural examination of the cell aggregates revealed cellular and extracellular morphology like that for a developing hypertrophic phenotype *in vivo* (Gerstenfeld and Landis, 1991). Formation of bone calvaria occurs mainly *via* a direct intramembranous and not *via* endochondral bone formation. To test osteogenic *versus* chondrogenic potential of cultured cells from calvaria, cells were obtained from 12-day chicken embryo calvariae after tissue condensation, but before extensive osteogenic differentiation, and from 17-day embryo calvariae when osteogenesis is well progressed. Cells from the younger embryos, but not from the older, showed chondrogenic differentiation as characterized by the expression of collagen type II and following a temporal progression of maturation by the expression of collagen type X. Cell populations from both ages of embryos showed progressive osteogenic differentiation, based on the expression of osteopontin, bone sialoprotein, and osteocalcin mRNAs. When the younger embryonic cultures were grown in conditions permissive for chondrogenesis, the number of chondrogenic cells increased from approximately 15 to approximately 50% of the population. Pulse labeling of the cultures with BrdU showed selective proliferation of the chondrogenic cells in comparison with the osteogenic cells (Toma et al., 1997).

MAPK signaling (ERK, JNK and p38) play a key role in the control of the chondrocyte phenotype. When in cultured primary bovine chondrocyte ERK and JNK were blocked using specific inhibitors, the expression of both chondrogenic and fibrotic marker genes increased (Rosenzweig et al., 2013). On the contrary, the blockade of p38 upregulated the expression of type II collagen but inhibited the expression of type I collagen suggesting a role of p38 in the dedifferentiation of chondrocytes under monolayer culture conditions. Inflammation may favor dedifferentiation. A pro-inflammatory cytokine, such as IL-1beta, promoted the dedifferentiation of cultured rabbit chondrocytes. NF-kB pathway, a prototypical proinflammatory signaling pathway, by inducing pathways eventually leading to the activation of ERK and p38, enhanced the effect of IL-1beta on the dedifferentiation of human chondrocytes *in vitro* (M. Li et al., 2019). A study by Hong and Reddi suggested that microRNAs are also involved in the control of chondrocyte phenotype. MicroRNA-221 and microRNA-222 were upregulated during dedifferentiation, while microRNA-140, microRNA-143, and microRNA-145 were downregulated (Hong and Reddi, 2013).

The freeing from the pre-existing matrix and the cell adherence to the culture dish are crucial for chondrocyte dedifferentiation. The Notch pathway regulated matrix metalloproteinase 13 (MMP-13) activity and the differentiation of human articular chondrocytes *in vitro* (Sassi et al., 2014). Integrin αvβ5 was reported to activate ERK signaling and to enhance the dedifferentiation of human articular chondrocytes (Fukui et al., 2011). The change of the actin cytoskeleton architecture, consequent to the cell adhesion and spreading, is also part of the regulation of the chondrocyte dedifferentiation (Parreno et al., 2017). The disruption of the actin cytoskeleton inhibited the dedifferentiation of cultured rabbit chondrocytes (Kim et al., 2003). Focal adhesion kinase (FAK) provides signaling at sites of integrin adhesion. The role of FAK in the chondrocyte dedifferentiation was investigated by Shin et al. A progressively higher level of focal adhesion complexes was observed with increased culture passages of rat chondrocytes. FAK knockdown promoted the restoration of cartilage-specific gene expression in the dedifferentiated chondrocytes (Shin et al., 2016). For a more detailed information on molecular mechanisms controlling chondrocyte dedifferentiation see (Yao and Wang, 2020).

The first surgical trial with autologous chondrocyte implantation (ACI) was performed in rabbit in 1989 (Grande et al., 1989) and the first pilot human study performed by Brittenberg et al. was published in 1994 (Brittberg et al., 1994). The first ACI treatments consisted of the implantation of autologous dedifferentiated chondrocytes into the damaged region under a periosteum flap or a synthetic membrane with an open joint procedure but, since then, the technology evolved to become an improved and worldwide well-established surgical technique. ACI was one of the first cell therapies approved by FDA and EMA regulatory agencies. Presently several commercial ventures are offering a service to expand autologous chondrocytes from harvested biopsies. For a recent review discussing the evolution of dedifferentiated articular chondrocyte implantation see (Davies and Kuiper, 2019). Since then, other cell sources with an *in vitro* proven chondrogenic potential, have been tested in human clinical trials for articular cartilage treatments including autologous nasal-septum derived chondrocytes (Mumme et al., 2016), autologous adipose MSCs (Jo et al., 2014), autologous bone-marrow (Nejadnik et al., 2010; Wakitani et al., 2011), allogeneic bone-marrow (Vega et al., 2015), and allogeneic Umbilical Cord Blood (Lim et al., 2021). A published study evaluated and compared the *in vitro* characteristics and chondrogenic capacity of some adult cells for use in cartilage repair, namely, bone-marrow MSCs, adipose MSCs, articular chondrocyte progenitors, and nasal septum-derived progenitors. Apparently, no major differences in the cell *in vitro* behavior were observed, although a preference was given to nasal septum-derived progenitors because of a slightly higher proliferation and chondrogenic potential and an easier access to the source tissue (Shafiee et al., 2016). However up to now autologous dedifferentiated chondrocytes remain the main source for the treatment of articular cartilage defect. The chondrocyte senescence, including the relatively low number of duplications these cells can perform *in vitro*, and the consequent growth arrest during the monolayer expansion, especially with chondrocytes derived from cartilage biopsies of older patients, remain a key factor that prevent to extend the treatment to the human population which, in principle, could benefit more from the cell implantation. Recently it has been reported that the supplementation of culture medium with platelet lysate induced the re-entry in the cell cycle of growth-arrested dedifferentiated chondrocyte thus making possible the *in vitro* expansion also of chondrocytes from cartilage biopsies of older patients (van Nguyen et al., 2018).

## Conclusion

The maintenance of tissue homeostasis and the tissue regeneration after an insult are fundamental functions of adult stem cells. The “stem cell niche” is the local micro-environment, defined by extracellular matrix, other neighborhood cells, and growth factor and cytokine signals, required for the maintenance of the cell stemness. Once stem cells leave their niche, they commit to a more restricted lineage leading into specific cell types. During this process stem cells give rise to TACs, an undifferentiated progenitor population in transition between SCs and post-mitotic differentiated cells. It is still debated whether cells from each SC division adopt divergent fates (i.e., one SC and one TAC) or if, when SCs double, they stochastically adopt SC or TAC fates. However, there is a consensus that TACs are not only a transitory phase from SCs to post-mitotic cells with the only role to generate differentiated cells, but they actively control proliferation and number of their ancestor SCs and proliferation and differentiation of their progeny toward tissue specific functional cells. Apparently, differences exist in different tissues. Autocrine signals and negative and positive feedback and feedforward paracrine signals play a major role in these controls. Although the expression of specific genes and the response to molecular signals can vary in TACs of different tissues, there are some genes and molecular pathways whose expression and activation is common to most TACs regardless than their tissue of origin. These include Wnt, Notch, Hedgehog and BMP pathways. The Wnt signal is crucial for inducing SC and TAC proliferation. In most tissues, activation of the Notch pathway keeps cells in a stem-cell state. On the contrary, when the Notch pathway is blocked, the cells differentiate precociously, even in the presence of a Wnt signal. Nevertheless, in some tissues Notch shows an opposite effect and drives the cells toward terminal differentiation. Hedgehog signaling is a key positive regulator of cell proliferation. BMPs promote cell differentiation and can block SC proliferation. We can expect that the bioinformatic and “omics” technologies available today, including single-cell transcriptomics, will allow, in a very near future, to define signatures of SCs, “early” and “late” TACs, and post-mitotic cells and to fully understand similarities and differences in mechanisms behind the transition from SCs to post-mitotic differentiated cells in each tissue.
